# Validation of a scale for assessing attitudes towards outcomes of genetic cancer testing among primary care providers and breast specialists

**DOI:** 10.1371/journal.pone.0178447

**Published:** 2017-06-01

**Authors:** Anne-Deborah Bouhnik, Khadim N’Diaye, D. Gareth Evans, Hilary Harris, Aad Tibben, Christi van Asperen, Joerg Schmidtke, Irmgard Nippert, Julien Mancini, Claire Julian-Reynier

**Affiliations:** 1 Aix Marseille Univ, INSERM, IRD, SESSTIM, Sciences Economiques & Sociales de la Santé & Traitement de l’Information Médicale, Marseille, France; 2 Department of Genomic Medicine, The University of Manchester, Manchester, United Kingdom; 3 GenEd Coordinating Centre, University of Manchester, Manchester, United Kingdom; 4 Department of Clinical Genetics, Leiden University Medical Centre, Leiden, The Netherlands; 5 Institute of Human Genetics, Hannover Medical School, Hannover, Germany; 6 Women’s Health Research, Münster Medical School, Münster, Germany; 7 APHM, Hôpital de la Timone, Service Biostatistique et Technologies de l’Information et de la Communication, Marseille, France; 8 Institut Paoli-Calmettes, UMR_S 912, Marseille, France; Ohio State University Wexner Medical Center, UNITED STATES

## Abstract

**Objective:**

To develop a generic scale for assessing attitudes towards genetic testing and to psychometrically assess these attitudes in the context of *BRCA1/2* among a sample of French general practitioners, breast specialists and gyneco-obstetricians.

**Study design and setting:**

Nested within the questionnaire developed for the European InCRisC (International Cancer Risk Communication Study) project were 14 items assessing expected benefits (8 items) and drawbacks (6 items) of the process of breast/ovarian genetic cancer testing (*BRCA1/2*). Another item assessed agreement with the statement that, overall, the expected health benefits of *BRCA1/2* testing exceeded its drawbacks, thereby justifying its prescription. The questionnaire was mailed to a sample of 1,852 French doctors. Of these, 182 breast specialists, 275 general practitioners and 294 gyneco-obstetricians completed and returned the questionnaire to the research team. Principal Component Analysis, Cronbach’s α coefficient, and Pearson’s correlation coefficients were used in the statistical analyses of collected data.

**Results:**

Three dimensions emerged from the respondents’ responses, and were classified under the headings: “Anxiety, Conflict and Discrimination”, “Risk Information”, and “Prevention and Surveillance”. Cronbach’s α coefficient for the 3 dimensions was 0.79, 0.76 and 0.62, respectively, and each dimension exhibited strong correlation with the overall indicator of agreement (criterion validity).

**Conclusions:**

The validation process of the 15 items regarding *BRCA1/2* testing revealed satisfactory psychometric properties for the creation of a new scale entitled the Attitudes Towards Genetic Testing for *BRCA1/2* (ATGT-*BRCA1/2*) Scale. Further testing is required to confirm the validity of this tool which could be used generically in other genetic contexts.

## Introduction

The first genes causing susceptibility to breast/ovarian cancer (*BRCA1/2* genes) were identified approximately twenty-five years ago [1]. Since then, *BRCA1/2* genetic testing has progressively become routine clinical practice in France [2] and in other industrialized countries [3–5]. A systematic review on risk assessment, genetic counselling, and genetic testing for *BRCA*-related cancer in women was recently performed to update the U.S. Preventive Services Task Force Recommendations (USPSTF) for care [6, 7]. One of the positive outcomes of *BRCA1/2* genetic cancer testing is that when a pathogenic germline variant has been identified, surveillance and preventive strategies can reduce breast/ovarian cancer incidence and increase life expectancy [7, 8]. However, while there is sufficient evidence to show the benefit of risk-reducing surgery for breast/ovarian cancer, evidence is lacking for intensive screening [9]. Women’s behaviors in terms of risk reduction and surveillance have been described up to 5 years after testing [10–12]. Results from different studies have highlighted the favorable impact of genetic testing, since the majority of those identified with *BRCA1/2* mutations in those studies adopted preventive strategies, including risk-reducing surgery and/or surveillance using imaging technologies. Another major outcome of a positive mutation test is that the risk of breast cancer running in a family can be assessed, that uncertainty regarding genetic risk can be removed, and strategies can be implemented to protect children and future generations [13].

Some studies have shown that among the other outcomes of *BRCA1/2* genetic cancer testing, breast cancer-related worry, anxiety and depression all increase in women testing positive, and decrease in those testing negative. These findings are not consistent across all the studies examined [7, 14]. One meta-analysis highlighted the importance of the time when such variables are measured, as the stress induced by a positive test result may decrease over time [15]. In some studies, risk perception improved after receiving test (positive or negative) results [7], and increased in those identified as *BRCA1/2* mutation carriers who had not had risk-reducing mastectomy 5 years after test results [10]. Other studies have pointed out the risks of familial conflict after the disclosure of results [16, 17], and the risk of misinterpretation of results by families [18]. From a legal point of view, many countries have enacted laws to protect individuals from discrimination, for example the Genetic Information Non-discrimination Act in the United States, and the Genetic Non-Discrimination Act in Canada. In France, a law was enacted in 2005 (2002–303 Art. L. 1141–1), which prohibits health and life insurance companies as well as employers from using genetic information based on an ‘analysis of genetic characteristics’ to the detriment of carriers. However, a study conducted among French unaffected *BRCA1/2* carriers pointed out that the risk of occupational discrimination may nonetheless exist [19]. The negative concerns highlighted above might counterbalance the positive outcomes of testing, resulting in a reduced desire by members of families who already have a history of breast cancer to go for testing.

In parallel, the attitude of primary care providers towards risk assessment and genetic testing has been described as a key determinant of how women approach testing and clinical management. One study showed that almost half of the women who considered clinical testing for *BRCA1/2* mutations wanted to have their primary doctor’s opinion first [20]. Other studies have highlighted disparities in the recommendations—largely attributable to physicians—provided to women as regards *BRCA1/2* testing [20–23]. The attitudes of primary care providers towards surveillance and prophylactic surgery have also been described as important in the management of identified *BRCA1/2* mutation carriers. A study among newly diagnosed breast cancer patients carrying a *BRCA1/2* mutation demonstrated that physicians’ recommendations influenced their decision to undergo a contralateral prophylactic mastectomy [24]. Moreover, one international study found considerable differences between different countries in physicians’ attitudes towards prophylactic mastectomy [23]. This heterogeneity in attitudes would suggest differences in the counselling provided to patients concerning prophylactic surgery and *BRCA1/2* genetic testing.

Since primary care providers refer high-risk populations to genetic cancer testing clinics, it is important to describe their overall assessment (i.e., positive or negative) of the testing process. To our knowledge, little information is currently available on this subject. Furthermore, no instruments have as yet been developed to evaluate each of the process’s specific expected benefits and drawbacks. Developing and validating such an instrument would enable international comparison, as well as the study of changes over time of stakeholders’ perceptions of genetic cancer testing.

Our primary objective therefore was to develop a generic scale for assessing attitudes towards genetic testing, and to psychometrically assess these attitudes in the context of *BRCA1/2* among a sample of French general practitioners, breast specialists and gyneco-obstetricians. Accordingly, we named the scale the “Attitudes Towards Genetic Testing for *BRCA1/2* (ATGT-*BRCA1/2*) Scale”.

## Materials and methods

### The InCRisC study

The International Cancer Risk Communication Study (InCRisC) is a multicenter European research project describing cancer risk communication practices and the management of familial breast cancer in primary care. It was carried out in 2010 in four European countries (France, Germany, the Netherlands and the UK). Questionnaires taking approximately 25 minutes to complete were mailed to a sample of general practitioners (GP) and breast specialists (BS) in each of these four countries. The French sample also included gyneco-obstetricians (GO) as they are very involved in patient referral for genetic cancer testing in France. The study’s methodology was reviewed and approved by the German Federal Ministry of Education and Research “Ethical, Legal and Social Implication of Biomedical Research” program, which also funded the project. A detailed description of the study design has been published elsewhere [23].

### French data

Only data from completed French questionnaires were considered for the present work. The list of French doctors for inclusion in InCRisC was generated using simple random sampling from the database of the technology and services company CEGEDIM (“Centre de Gestion, de Documentation, d'Informatique et de Marketing”): GP (N = 750), BS (N = 352) and GO (N = 750). Practitioners who did not have at least one encounter with a breast cancer patient during the year prior to the survey were excluded from the analysis.

### Questionnaire

The InCRisC questionnaire collected data on personal (gender, age) and occupational characteristics (number of years working in specialized field, number of medical students in practice, area of practice). GP were also asked approximately how many patients consulted them per week, while BS and GO were asked about the number of newly diagnosed breast cancer patients who had consulted them during the previous year.

Nested in the questionnaire were 15 items, developed by international experts in the *BRCA1/2* genetic testing field who are all co-authors of this manuscript (GE, HH, AT, CA, JS, IN, CJR), to evaluate attitudes towards *BRCA1/2* genetic testing. The first 14 assessed positive (8 items) and negative (6 items) expected outcomes of *BRCA1/2* genetic testing. The last assessed overall agreement with the statement that the expected health benefits of *BRCA1/2* testing exceeded the drawbacks, thereby justifying its prescription (Table 1). All 14 positive/negative items were worded in such a way that they could be answered by practitioners, patients and the general population. Only one item (number 7) was worded specifically for *BRCA1/2* genetic testing. These choices in wording were made in order for these items to be easily reusable in other genetic testing contexts.

**Table 1:** Descriptive statistics of the items regarding attitudes towards *BRCA1/2* genetic testing (InCRisC France, n = 751).

Item	Mean	SD	Missing n (%)	Not at all beneficial (0) n (%)	Slightly beneficial (1) n (%)	Somewhat beneficial (2) n (%)	Beneficial (3) n (%)	Very beneficial (4) n (%)
**In your opinion what are the potential benefits of predictive genetic testing for an unaffected patient in whose family a *BRCA1* or *BRCA2* mutation has been identified?**								
1. Removal of uncertainty of genetic risk	2.98	0.85	38 (5.1)	8 (1.1)	31 (4.1)	124 (16.5)	357 (47.5)	193 (25.7)
2. Provision of information for children	3.09	0.71	29 (3.9)	0 (0.0)	19 (2.5)	94 (12.5)	411 (54.7)	198 (26.4)
3. Provision of information for other family members	2.95	0.80	31 (4.1)	7 (0.9)	31 (4.1)	115 (15.3)	404 (53.8)	163 (21.7)
4. Provision of information for family planning	2.57	1.04	34 (4.5)	30 (4.0)	82 (10.9)	187 (24.9)	288 (38.3)	130 (17.3)
5. Access to increased surveillance and screening	3.49	0.64	32 (4. 3)	4 (0.5)	2 (0.3)	29 (3.9)	285 (37.9)	399 (53.1)
6. Access to clinical trials	2.36	1.01	49 (6.5)	35 (4.7)	93 (12.4)	233 (31.0)	264 (35.2)	77 (10.3)
7. Greater choices of risk-reducing management options* for patients with a positive test result	2.88	0.95	32 (4.3)	21 (2.8)	35 (4.7)	139 (18.5)	337 (44.9)	187 (24.9)
8. Potential increase of life expectancy due to increased surveillance	3.31	0.75	27 (3.6)	7 (0.9)	8 (1.1)	59 (7.9)	333 (44.3)	317 (42.2)
				**Very low/none (0)** **n(%)**	**Low (1)** **n(%)**	**Moderate (2)** **n (%)**	**High (3)** **n (%)**	**Very high (4)** **n (%)**
**In your opinion the likelihood of the following negative consequences of predictive *BRCA1* and *BRCA2* testing are …?**								
9. Psychological distress about being identified positive due to the documented increased risk of breast cancer	3.24	0.69	16 (2.1)	4 (0.5)	4 (0.5)	73 (9.7)	385 (51.3)	269 (35.8)
10. Family conflict about disclosure of test results	2.00	1.01	21 (2.8)	61 (8.1)	144 (19.2)	304 (40.5)	177 (23.6)	44 (5.9)
**In your opinion what is the likelihood of potential abuse / misuse of a positive *BRCA1* or *BRCA2* test result by third parties which would result in…**								
11. …limited employment opportunities	1.87	1.22	35 (4.7)	136 (18.1)	126 (16.8)	201 (26.8)	201 (26.8)	52 (6.9)
12. …limited life insurance coverage	2.69	1.18	34 (4.5)	66 (8.8)	42 (5.6)	122 (16.2)	308 (41.0)	179 (23.8)
13. …limited private health insurance coverage	2.25	1.28	35 (4.7)	107 (14.2)	83 (11.1)	169 (22.5)	240 (32.0)	117 (15.6)
14. …stigmatization as a “healthy ill” person	2.21	1.20	30 (4.0)	90 (12.0)	91 (12.1)	214 (28.5)	232 (30.9)	94 (12.5)
				**Strongly disagree (0)** **n(%)**	**Disagree (1)** **n (%)**	**Agree (2)** **n (%)**	**Strongly agree(3)** **n (%)**	
**15.Do you agree or disagree with the following statement:**								
The expected health benefits of *BRCA1* and *BRCA2* testing exceed expected negative consequences by a sufficiently wide margin to justify prescribing the test	2.18	0.67	25 (3.3)	14 (1.9)	69 (9.2)	414 (55.1)	229 (30.5)	

* prophylactic mastectomy/oophorectomy

### Statistical analysis

#### Principal component analysis

Principal Component Analysis was first carried out on data from doctors who answered all 14 positive/negative items. The aim of this analysis was to investigate the patterns underlying doctors’ responses to the items and extract independent dimensions that might explain most of the variance in the sample.

In line with Kaiser’s criterion, only dimensions with eigenvalues >1 were selected.

A factorial analysis with an orthogonal rotation (Varimax) was performed to simplify the interpretation of the results obtained for the selected dimensions. The rates of variance explained by them were determined and factor loading values verified. Only items with a factor loading >|0.40| on a single dimension were selected for inclusion in the new scale.

#### Scale quality criteria

Dimensions were selected using Principal Component Analysis. Each was associated with a different number of the questionnaire’s 14 positive/negative items and was used to define a sub-scale. The score obtained for each sub-scale was computed by summing the answers to the particular items associated with that dimension: for the 8 positive benefit items (items 1 to 8), scores for answers were as follows: not at all beneficial = 0; slightly beneficial = 1; somewhat beneficial = 2; beneficial = 3; very beneficial = 4.

For the 6 negative consequence items (items 9 to 14), scores were as follows: very low/none (likelihood) = 0; low = 1; moderate = 2; high = 3; very high = 4).

Total scores for each dimension were linearly transformed to range from 0 to 100.

In each sub-scale, the floor and ceiling effects, defined as the percentage of subjects with the lowest and highest possible scores, respectively, were examined and compared with the 15% threshold described as causing an abnormal score distribution [25].

The Internal Consistency method was used to assess the reliability of each sub-scale, and Cronbach’s α coefficient was computed to establish the internal consistency of each set of items. For each sub-scale, in order to avoid redundancy, items for which the coefficient increased when they were removed were eliminated.

Pearson’s correlation coefficients between the dimensions were computed for each possible pairing of the selected dimensions. In each case, the correlation coefficient was then compared with the two associated Cronbach’s α coefficients. If the correlation coefficient was lower than the Cronbach’s α value, the dimensions were considered to measure different aspects [26, 27].

The construct-related validity was determined by assessing each item’s convergent validity (the correlation between an item and the dimension to which it belonged had to be greater than 0.40) and its discriminant validity (the correlation between an item and the dimension to which it belonged had to be greater than that obtained for the item and the other dimensions).

To determine the ability of the overall new scale (i.e. the sub-scales together) to discriminate between doctors in terms of their attitudes to genetic testing and characteristics (gender, age and specialty), comparisons were made with the overall indicator of agreement item (i.e., that benefits of BRCA1/2 testing exceeded the drawbacks). Criterion validity comparisons were made by performing analyses of variance and linear regressions, taking p-values of less than 0.05 to be statistically significant. The software package SPSS/PC version 18.0 was used to perform various analyses.

## Results

### Sample characteristics

Of the 1852 French doctors contacted by mail, a total of 751 (275 GP, 182 BS and 294 GO) were included, giving an overall response rate of 40.5% (36.7%, 51.7% and 39.2% among GP, BS and GO, respectively). GP and BS comprised mostly men (62.9% and 88.5%, respectively) while GO comprised mostly women (63.9%), p<0.001. Mean age was comparable between BS and GO (52.3 years and 51.9 years, respectively), while GP were slightly younger (48.5 years, p<0.001).

### Description of the characteristics of the 14 scale-specific positive/negative questionnaire items

A description of the 14 scale-specific positive/negative questionnaire items is provided in Table 1. Missing data accounted for 2.1% (“Psychological distress from being identified as a mutation carrier, due to increased risk of breast cancer”) to 6.5% (“Access to clinical trials”) of answers. Mean scores were all above 2 (possible range 0 to 4), except for item 11 (“Potential abuse of results by third party resulting in limited employment opportunities”) whose mean score was 1.87. The highest score was 3.49 for item 5 “Access to increased surveillance and screening”.

In the subsequent analyses, the study population was reduced to the 659 doctors (87.7%) who answered all 14 positive/negative items. These 659 doctors included a higher proportion of GP (38.5%) and a lower proportion of GO (37.8%) than the proportion of doctors (n = 92) who did not answer all the items (GP: 22.8%, GO: 48.9%, p = 0.01). No other difference was found between these two groups and no difference in the proportions of respondents and non-respondents was observed for BS.

### Principal component analysis

Principal Component Analysis was conducted on the 659 doctors’ answers to the 14 positive/negative items. Three independent dimensions with eigenvalues > 1 were identified and selected. The various questionnaire items loaded onto each dimension had a loading factor > |0.40|. The three dimensions selected explained 53.4% of the variance (Table 2).

**Table 2:** Factor loading resulting from Principal Component Analysis (varimax rotation) on the 14 items comprising the scale (InCRisC France, n = 659).

	Factors			Pearson correlations for convergent and discriminant validity		
Item	1	2	3	Dimension 1	Dimension 2	Dimension 3
1. Removal of uncertainty of genetic risk	-0.02	**0.67**	0.18	0.03	**-0.73**	-0.29
2. Provision of information for children	0.07	**0.79**	0.28	0.10	**-0.82**	-0.39
3. Provision of information for other family members	0.04	**0.78**	0.20	0.06	**-0.79**	-0.34
4. Provision of information for family planning	-0.05	**0.57**	0.38	0.08	**-0.76**	-0.42
5. Access to increased surveillance and screening	0.04	0.18	**0.70**	0.01	-0.33	**-0.67**
6. Access to clinical trials	-0.05	0.22	**0.43**	-0.03	-0.26	**-0.68**
7. Greater choice of risk-reduction management	-0.01	0.22	**0.63**	-0.01	-0.37	**-0.71**
8. Potential increase of life expectancy thanks to increased surveillance	0.09	0.18	**0.73**	0.05	-0.35	**-0.72**
9. Psychological distress at being identified as a mutation carrier, due to increased risk of breast cancer	**0.45**	0.34	-0.38	**0.50**	-0.07	0.04
10. Family conflict about disclosure of test results	**0.51**	0.31	-0.28	**0.57**	-0.06	0.01
11. …limited employment opportunities	**0.76**	0.00	0.05	**0.75**	-0.04	-0.02
12. …limited life insurance coverage	**0.82**	-0.09	0.09	**0.79**	-0.02	0.00
13. …limited private health insurance coverage	**0.78**	-0.08	0.14	**0.76**	-0.05	-0.03
14. …stigmatization as a “healthy ill” person	**0.78**	0.06	-0.08	**0.78**	-0.03	0.03
Eigenvalue	2.93	2.40	2.15			
% variance	20.94	17.11	15.33			

### Quality criteria

The first sub-scale identified included items 9 to 14 which corresponded to expected drawbacks of genetic testing. This sub-scale was labelled **“Anxiety, conflict and discrimination”.**

Cronbach’s α for this sub-scale was 0.79 (Table 3). The score obtained by summing replies to all 6 items, and transformed in order to obtain a range of values from 0 to 100, yielded a mean score of 59.7 (SD = 19.4). The observed range of values covered all the range of possible values.

**Table 3:** Descriptive data and reliability coefficients of the three dimensions of participants’ attitudes towards the *BRCA1/2* genetic testing scale–(InCRisC France, n = 659).

	Subscale 1 Anxiety-conflict and discrimination	Subscale 2 Risk Information	Subscale 3 Prevention-Surveillance	Overall scale Attitudes Towards Gene Testing for *BRCA1/2*
**Cronbach’s alpha**	0.79	0.76	0.62	0.73
**Score (mean ± SD)**	59.67±19.39	72.86±16.28	75.35±14.56	57.22±11.92
**Median Value**	62.5	75.0	75.0	56.8
**Possible score range**	0–100	0–100	0–100	0–100
**Observed score range**	0–100	18.8–100	12.5–100	19.8.3–95.3
**Floor n (%)**	2 (0.30)	0 (0.00)	0 (0.00)	0 (0.00)
**Ceiling, n (%)**	10 (1.52)	80 (12.14)	44 (6.68)	0 (0.00)
**The expected health benefits of BRCA1 and BRCA2 testing exceed anticipated negative consequences by a sufficiently wide margin to justify prescribing the test:**	mean (SD)	mean (SD)	mean (SD)	mean (SD)
Strongly disagree / Disagree	69.31 (19.03)	61.80 (19.69)	67.52 (17.54)	47.67 (12.91)
Agree	60.00 (17.94)	71.03 (15.05)	73.32 (13.35)	56.09 (10.65)
Strongly agree	55.61 (21.04)	80.02 (14.30)	82.12 (12.70)	62.73 (11.16)
P-value	<0.001	<0.001	<0.001	<0.001

The second sub-scale included items 1 to 4 which corresponded to expected benefits and was labelled **“Risk Information”**. The scores calculated for this second dimension ranged from 0 to 100, with a mean score of 72.9 (SD = 16.3). Observed values ranged from 18.8 to 100. Cronbach’s α for this second sub-scale was 0.76.

The third sub-scale was labelled “**Prevention-Surveillance**”, and included items 5 to 8 which also corresponded to expected benefits. These scores also ranged from 0 to 100. Mean score was 75.4 (SD = 14.6), and observed values ranged from 12.5 to 100. Cronbach’s α for this third sub-scale was 0.62.

No floor or ceiling effects beyond the 15% threshold were observed for the three sub-scales, and each inter-scale correlation coefficient was lower than the corresponding Cronbach’s α (Tables 2 and 3). In each of the three dimensions, all items met both convergent validity and discriminant validity criteria.

The new scale which we created included all 14 positive/negative items described and analyzed above. We named it the **“Attitudes Towards Gene Testing for *BRCA1/2*” (ATGT-*BRCA1/2*) Scale”**. The overall score obtained for this scale was a linear combination of the three sub-scores, each one contributing equally [28], and transformed to obtain a range of values between 0 and 100 (i.e., (subscale scores 2+3—subscale score 1 +100)/300). An overall positive score denoted an overall positive attitude towards *BRCA1/2* genetic testing. The observed values of the score ranged from 19.8 to 95.3, with a mean value of 57.2 (SD = 11.9). No floor or ceiling effects were observed and Cronbach’s α was 0.73.

### Criterion validity

**Fig 1:**
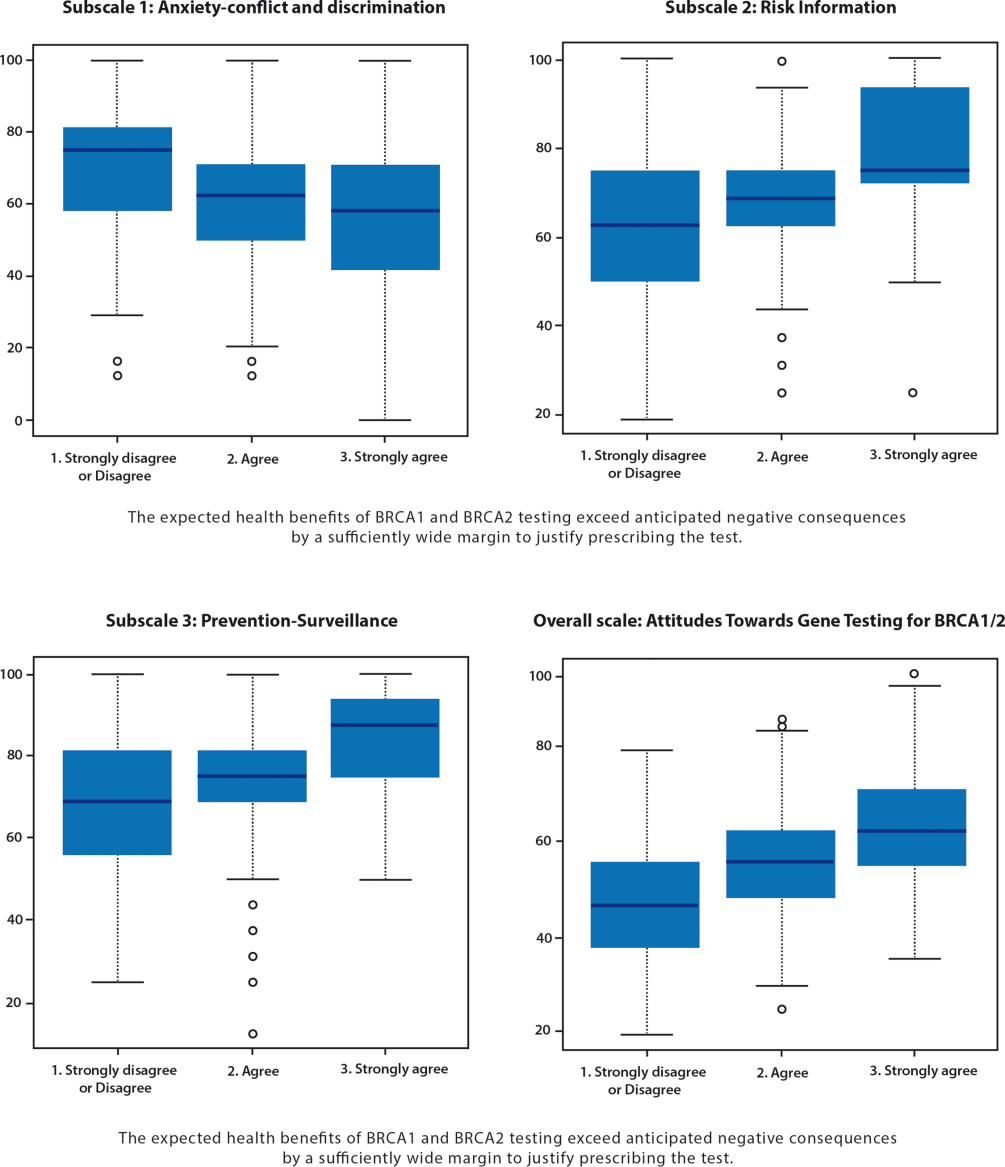
Distribution of the three subscales and of the overall scale by the overall indicator–(InCRisC France, n = 659).

Fifty-five point one percent and 30.5% of doctors, agreed and strongly agreed, respectively, with the statement that the expected health benefits of *BRCA1/2* genetic testing exceeded expected drawbacks by a sufficiently wide margin to justify prescribing the test (Table 1). Only 11.1% disagreed or strongly disagreed with this statement. As shown in Table 3 and in Fig 1, this overall indicator was strongly associated with all three sub-scales, as well as with the overall scale.

Finally, when comparing scores according to physicians’ characteristics, male physicians scored lower on the first dimension, as well as breast surgeons (Table 4). By contrast, breast surgeons scored higher on the third dimension, as well as on the overall scale. No characteristics were associated with the second dimension.

**Table 4:** Factors associated with the three scores and the overall score of attitude towards *BRCA1/2* gene testing–(InCRisC France, n = 659).

Univariate analyses								
	Score 1		Score 2		Score 3		Overall score	
	**Mean (SD)**	**P-value**	**Mean (SD)**	**P-value**	**Mean (SD)**	**P-value**	**Mean (SD)**	**P-value**
**Gender**		0.004		0.66		0.67		0.02
Female	62.29 (18.84)		72.53 (16.31)		75.64 (14.31)		55.90 (11.92)	
Male	13.88 (19.58)		73.09 (16.28)		75.15 (14.76)		58.15 (11.84)	
**Age**	-0.06*	0.11	0.05*	0.25	-0.01*	0.71	0.06*	0.11
**Specialty**		0.03		0.21		0.09		0.02
General Practitioner	60.78 (18.54)		71.60 (16.54)		74.75 (13.73)		56.20 (11.21)	
Breast surgeon	56.01 (20.89)		72.80 (16.08)		77.60 (15.70)		59.60 (12.68)	
Gyneco-obstetrician	60.84 (19.05)		74.17 (16.11)		74.55 (14.56)		56.76 (11.97)	
**Multivariate analyses**								
	**Β (SE)**	**P-value**	**Β (SE)**	**P-value**	**Β (SE)**	**P-value**	**Β (SE)**	**P-value**
**Gender**								
Female	Réf							
Male	-3.68 (1.66)	0.03						
**Age**								
**Specialty**								
General Practitioner	Réf				Réf		Réf	
Breast surgeon	-3.85 (2.00)	0.05			2.85 (1.48)	0.05	3.39 (1.21)	0.01
Gyneco-obstetrician	-0.86 (1.77)	0.63			-0.21 (1.30)	0.87	0.56 (1.06)	0.60

* Pearson’s correlation coefficient.

## Discussion

*BRCA1/2* genetic cancer testing helps to reduce breast/ovarian cancer incidence and increase life expectancy [7, 8]. However, concerns regarding anxiety, distress, familial conflict and/or discrimination [7, 14–17, 19] can influence the decision to go for testing, especially in individuals from families with a history of breast cancer. This decision may also be influenced by physicians’ attitudes to testing, as stressed by several studies [20–24].These considerations highlight the importance of documenting patients’ and physicians’ attitudes to testing, especially those of primary care providers as they are the professionals who refer high-risk populations to genetic cancer testing clinics.

In the development of the new **Attitudes Towards Gene Testing for *BRCA1/2* (ATGT-*BRCA1/2*) Scale** for assessing positive and negative outcomes of *BRCA1/2* genetic testing, three dimensions were identified. One reflected the expected drawbacks of genetic testing and two the expected benefits (a family-based dimension and a medical management dimension). The overall scale score combined perceptions towards both positive and negative outcomes of genetic testing. The three dimensions examined each correlated with the overall indicator. The reliability coefficients were above 0.7 for the first and second subscales, as well as for the overall indicator. This threshold of 0.7 is commonly recommended as being indicative of sufficient reliability [29]. However, in exploratory research, the acceptable threshold may be lowered to 0.6 [30–34], which means that in the present work the third subscale can also be considered as reliable. Globally, the psychometric properties of this scale were satisfactory in terms of construct validity, internal consistency and criterion validity, making it a valid tool to measure attitudes towards genetic testing. The rates of missing data were low. The highest missing data rate—6.5% for item 6 (positive) regarding “Access to clinical trials”—can be explained by the fact that clinical trials cover a range of different contexts depending on the type of trial (phase I, phase II or phase III) and on what is being evaluated [35]. Accordingly, the expected benefits differ from one trial to the next, and inclusion does not necessarily represent hope for the patient (for example, situations where phase I trials are proposed to patients because there is no alternative treatment to offer them). Consequently, people may therefore find it more difficult to evaluate this item.

Few questionnaires on genetic testing currently exist. Examples are the MICRA scale (Multidimensional Impact of Cancer Risk Assessment), which evaluates the impact of disclosure after genetic testing [36], and the PAHC questionnaire (Psychosocial Aspects of Hereditary Cancer) [37], designed to assess problems related to cancer genetic counseling. The Impact of Event Scale (IES) mainly focuses on psychological distress [38]. Moreover, these scales target specific patients. On the contrary, our new scale was conceived to be as generic as possible, and may be used for different populations, such as patients, caregivers, healthcare providers, as well as individuals in the general population. This gives it a major advantage as attitudes may be directly compared between different populations.

Another advantage of this new scale is that its items are not specific to *BRCA1/2* genetic testing (except for item 7 which refers to prophylactic surgery). This was a design choice by the developers in order to make the scale suitable for use in other genetic contexts, such as *MLH1* or *MSH2*, for colorectal cancer, but also for non-cancer related contexts. It may also be adapted to new genetic testing processes. As pointed out above, only the labelling of item 7 would need to be adapted to the specific context.

In our population of professional caregivers, the median score of the overall scale was over 50 (out of 100), which shows that *BRCA1/2* genetic testing was, overall, considered positively by French physicians, especially by breast surgeons. This was consistent with the response to item 15 of the questionnaire (i.e., the overall indicator). This result may be a little surprising since French care providers were identified as less favorable to prophylactic mastectomy than their UK, German and Dutch counterparts [23]. However, this fact might be explained by the strong probability that the latter would provide an even higher score on this scale were they to answer the same 14 items, and also because this assessment explores several different aspects of genetic testing (i.e., more than just medical management aspects like prophylactic mastectomy), thereby reflecting a more comprehensive perspective by French physicians. Accordingly, the differences between countries might not be so large. The next step would be to validate and compare the scores of physicians of the other European countries who took part in the InCRisC study.

It would also be interesting to compare the attitudes of physicians with those of patients who have *BRCA1/2* gene mutations. One may hypothesize that the latter would have higher positive scores on this scale, but also higher negative scores given that it is they who physically undergo the test.

Studying changes over time in attitudes to testing among *BRCA1/2* mutation carriers but also among physicians and the general population might also be an interesting perspective. The new tool proposed here might also be sensitive to possible future evolutions in attitudes, either positive or negative, as genetic testing techniques are improved.

Moreover, a future step in the implementation of this tool would be to determine informative thresholds for each dimension which could then be used when applying this scale. This would help evaluate which of the three dimensions would most determine an individual’s overall attitude towards genetic testing.

Some study limitations have to be acknowledged.

First, the questionnaire response rate obtained was low despite the reminders made by mail and telephone. It is very probable that a large proportion of the physicians who agreed to answer the questionnaire had extreme positive or negative opinions towards genetic testing. Therefore, the low response rate certainly reduces the possibility of generalizing the results, as it lacks the views of physicians with moderate opinions. Second, validation was performed on the physicians who answered all 14 positive/negative items. Comparisons with physicians who did not answer all 14 items showed that the proportion of GO in the former group was higher. Moreover, it remains uncertain if a net positive score on our scale can be translated into actual endorsement of genetic testing and/or referral of patients for genetic counseling or testing. This will have to be analyzed in further studies. Finally, before using this instrument in other settings, further research should be performed to validate this three-dimensional scale in other populations, in *BRCA1/2* carriers in particular, and in genetic contexts outside *BRCA1/2*. Despite these limitations, this new tool provides a novel means to measure physicians’ assessment of the expected benefits and drawbacks of *BRCA1/2* genetic cancer testing.

Further studies are required to assess the possibility of extending the present results to other contexts. The scale we developed may be adapted to other genetic contexts, and administered to other sub-populations, patients, providers and general populations. The work described here is the first step in the validation of this scale. Further research is now required to confirm the validity of the findings obtained for the three subscales by applying this tool to other populations in other settings.

## Supporting information

S1 File Data set. (XLSX)

## References

[1] Couch FJ, Nathanson KL, Offit K. Two decades after BRCA: setting paradigms in personalized cancer care and prevention. Science. 2014;343(6178):1466–70. PubMed Central PMCID: PMCPMC4074902. doi: 10.1126/science.1251827 24675953 10.1126/science.1251827 PMC4074902

[2] INCA. Oncogénétique en 2014—Consultations, laboratoires & prise en charge. décision cAàl, editor. Boulogne-Billancourt2016.

[3] Armstrong J, Toscano M, Kotchko N, Friedman S, Schwartz MD, Virgo KS, et al Utilization and Outcomes of BRCA Genetic Testing and Counseling in a National Commercially Insured Population: The ABOUT Study. JAMA Oncol. 2015;1(9):1251–60. doi: 10.1001/jamaoncol.2015.3048 26426480 10.1001/jamaoncol.2015.3048

[4] Slade I, Riddell D, Turnbull C, Hanson H, Rahman N, programme MCG. Development of cancer genetic services in the UK: A national consultation. Genome Med. 2015;7(1):18 PubMed Central PMCID: PMCPMC4341881. doi: 10.1186/s13073-015-0128-4 25722743 10.1186/s13073-015-0128-4 PMC4341881

[5] Vig HS, Wang C. The evolution of personalized cancer genetic counseling in the era of personalized medicine. Fam Cancer. 2012;11(3):539–44. PubMed Central PMCID: PMCPMC3905734. doi: 10.1007/s10689-012-9524-8 22419176 10.1007/s10689-012-9524-8 PMC3905734

[6] Nelson HD, Fu R, Goddard K, Mitchell JP, Okinaka-Hu L, Pappas M, et al. Risk Assessment, Genetic Counseling, and Genetic Testing for BRCA-Related Cancer: Systematic Review to Update the US Preventive Services Task Force Recommendation. U.S. Preventive Services Task Force Evidence Syntheses, formerly Systematic Evidence Reviews. Rockville (MD)2013.

[7] Nelson HD, Pappas M, Zakher B, Mitchell JP, Okinaka-Hu L, Fu R. Risk assessment, genetic counseling, and genetic testing for BRCA-related cancer in women: a systematic review to update the U.S. Preventive Services Task Force recommendation. Ann Intern Med. 2014;160(4):255–66. doi: 10.7326/M13-1684 24366442 10.7326/M13-1684

[8] Nathanson KL, Domchek SM. Therapeutic approaches for women predisposed to breast cancer. Annu Rev Med. 2011;62:295–306. doi: 10.1146/annurev-med-010910-110221 21034216 10.1146/annurev-med-010910-110221

[9] Moyer VA, Force USPST. Risk assessment, genetic counseling, and genetic testing for BRCA-related cancer in women: U.S. Preventive Services Task Force recommendation statement. Ann Intern Med. 2014;160(4):271–81. doi: 10.7326/M13-2747 24366376 10.7326/M13-2747

[10] Julian-Reynier C, Mancini J, Mouret-Fourme E, Gauthier-Villars M, Bonadona V, Berthet P, et al Cancer risk management strategies and perceptions of unaffected women 5 years after predictive genetic testing for BRCA1/2 mutations. Eur J Hum Genet. 2011;19(5):500–6. PubMed Central PMCID: PMCPMC3083622. doi: 10.1038/ejhg.2010.241 21267012 10.1038/ejhg.2010.241 PMC3083622

[11] Schwartz MD, Isaacs C, Graves KD, Poggi E, Peshkin BN, Gell C, et al Long-term outcomes of BRCA1/BRCA2 testing: risk reduction and surveillance. Cancer. 2012;118(2):510–7. PubMed Central PMCID: PMCPMC3286617. doi: 10.1002/cncr.26294 21717445 10.1002/cncr.26294 PMC3286617

[12] Gopie JP, ter Kuile MM, Timman R, Mureau MA, Tibben A. Impact of delayed implant and DIEP flap breast reconstruction on body image and sexual satisfaction: a prospective follow-up study. Psychooncology. 2014;23(1):100–7. doi: 10.1002/pon.3377 23983109 10.1002/pon.3377

[13] Julian-Reynier C, Fabre R, Coupier I, Stoppa-Lyonnet D, Lasset C, Caron O, et al BRCA1/2 carriers: their childbearing plans and theoretical intentions about having preimplantation genetic diagnosis and prenatal diagnosis. Genet Med. 2012;14(5):527–34. PubMed Central PMCID: PMCPMC4088944. doi: 10.1038/gim.2011.27 22241105 10.1038/gim.2011.27 PMC4088944

[14] Graves KD, Vegella P, Poggi EA, Peshkin BN, Tong A, Isaacs C, et al Long-term psychosocial outcomes of BRCA1/BRCA2 testing: differences across affected status and risk-reducing surgery choice. Cancer Epidemiol Biomarkers Prev. 2012;21(3):445–55. PubMed Central PMCID: PMCPMC3297701. doi: 10.1158/1055-9965.EPI-11-0991 22328347 10.1158/1055-9965.EPI-11-0991 PMC3297701

[15] Hamilton JG, Lobel M, Moyer A. Emotional distress following genetic testing for hereditary breast and ovarian cancer: a meta-analytic review. Health Psychol. 2009;28(4):510–8. PubMed Central PMCID: PMCPMC2807362. doi: 10.1037/a0014778 19594276 10.1037/a0014778 PMC2807362

[16] Douglas HA, Hamilton RJ, Grubs RE. The effect of BRCA gene testing on family relationships: A thematic analysis of qualitative interviews. J Genet Couns. 2009;18(5):418–35. doi: 10.1007/s10897-009-9232-1 19479365 10.1007/s10897-009-9232-1

[17] Lapointe J, Bouchard K, Patenaude AF, Maunsell E, Simard J, Dorval M. Incidence and predictors of positive and negative effects of BRCA1/2 genetic testing on familial relationships: a 3-year follow-up study. Genet Med. 2012;14(1):60–8. doi: 10.1038/gim.0b013e3182310a7f 22237432 10.1038/gim.0b013e3182310a7f

[18] Vos J, Menko F, Jansen AM, van Asperen CJ, Stiggelbout AM, Tibben A. A whisper-game perspective on the family communication of DNA-test results: a retrospective study on the communication process of BRCA1/2-test results between proband and relatives. Fam Cancer. 2011;10(1):87–96. PubMed Central PMCID: PMCPMC3036814. doi: 10.1007/s10689-010-9385-y 20852944 10.1007/s10689-010-9385-y PMC3036814

[19] Eisinger F, Fabre R, Lasset C, Stoppa-Lyonnet D, Julian-Reynier C, Nogues C. Spontaneous disclosure of BRCA1/2 genetic test results to employers: a French prospective study. Eur J Hum Genet. 2012;20(9):981–3. PubMed Central PMCID: PMCPMC3421122. doi: 10.1038/ejhg.2012.37 22378286 10.1038/ejhg.2012.37 PMC3421122

[20] Armstrong K, Stopfer J, Calzone K, Fitzgerald G, Coyne J, Weber B. What does my doctor think? Preferences for knowing the doctor's opinion among women considering clinical testing for BRCA1/2 mutations. Genet Test. 2002;6(2):115–8. doi: 10.1089/10906570260199366 12215250 10.1089/10906570260199366

[21] McCarthy AM, Bristol M, Domchek SM, Groeneveld PW, Kim Y, Motanya UN, et al Health Care Segregation, Physician Recommendation, and Racial Disparities in BRCA1/2 Testing Among Women With Breast Cancer. J Clin Oncol. 2016;34(22):2610–8. doi: 10.1200/JCO.2015.66.0019 27161971 10.1200/JCO.2015.66.0019 PMC5012689

[22] McCarthy AM, Bristol M, Fredricks T, Wilkins L, Roelfsema I, Liao K, et al Are physician recommendations for BRCA1/2 testing in patients with breast cancer appropriate? A population-based study. Cancer. 2013;119(20):3596–603. PubMed Central PMCID: PMCPMC3795950. doi: 10.1002/cncr.28268 23861169 10.1002/cncr.28268 PMC3795950

[23] Den Heijer M, van Asperen CJ, Harris H, Nippert I, Schmidtke J, Bouhnik AD, et al International variation in physicians' attitudes towards prophylactic mastectomy—comparison between France, Germany, the Netherlands and the United Kingdom. Eur J Cancer. 2013;49(13):2798–805. doi: 10.1016/j.ejca.2013.04.025 23692813 10.1016/j.ejca.2013.04.025

[24] Schwartz MD, Lerman C, Brogan B, Peshkin BN, Halbert CH, DeMarco T, et al Impact of BRCA1/BRCA2 counseling and testing on newly diagnosed breast cancer patients. J Clin Oncol. 2004;22(10):1823–9. doi: 10.1200/JCO.2004.04.086 15067026 10.1200/JCO.2004.04.086

[25] Terwee CB, Bot SD, de Boer MR, van der Windt DA, Knol DL, Dekker J, et al Quality criteria were proposed for measurement properties of health status questionnaires. J Clin Epidemiol. 2007;60(1):34–42. doi: 10.1016/j.jclinepi.2006.03.012 17161752 10.1016/j.jclinepi.2006.03.012

[26] Guilford JP. Psychometric methods New York: McGraw-Hill; 1954.

[27] Ware JE Jr., Gandek B. Methods for testing data quality, scaling assumptions, and reliability: the IQOLA Project approach. International Quality of Life Assessment. J Clin Epidemiol. 1998;51(11):945–52. 9817111 10.1016/s0895-4356(98)00085-7

[28] Streiner DL, Norman GR. Health measurement scales: a practical guide to their development and use: Oxford University Press; 1995.

[29] Nunnally JC. Psychometric theory: McGraw-Hill; 1978.

[30] Bowling A. Research Methods In Health: Investigating Health and Health Services: McGraw-Hill Education; 2009.

[31] Norman GR, Streiner DL. Biostatistics: The Bare Essentials: B.C. Decker; 2008.

[32] Nunnally J, Bernstein I. Psychometric theory. 3rd ed. New-York: McGraw-Hill; 1994.

[33] Robinson JP, Shaver PR, Wrightsman LS, Andrews FM. Measures of Personality and Social Psychological Attitudes: Academic Press; 1991.

[34] Streiner D, Norman G. Health Measurement Scales. New York: Oxford; 2000 Nov.

[35] Djulbegovic B, Kumar A, Glasziou P, Miladinovic B, Chalmers I. Medical research: Trial unpredictability yields predictable therapy gains. Nature. 2013;500(7463):395–6. PubMed Central PMCID: PMCPMC3819120. doi: 10.1038/500395a 23969443 10.1038/500395a PMC3819120

[36] Cella D, Hughes C, Peterman A, Chang CH, Peshkin BN, Schwartz MD, et al A brief assessment of concerns associated with genetic testing for cancer: the Multidimensional Impact of Cancer Risk Assessment (MICRA) questionnaire. Health Psychol. 2002;21(6):564–72. 12433008

[37] Eijzenga W, Bleiker EM, Hahn DE, Kluijt I, Sidharta GN, Gundy C, et al Psychosocial aspects of hereditary cancer (PAHC) questionnaire: development and testing of a screening questionnaire for use in clinical cancer genetics. Psychooncology. 2014;23(8):862–9. doi: 10.1002/pon.3485 24443031 10.1002/pon.3485

[38] Horowitz M, Wilner N, Alvarez W. Impact of Event Scale: a measure of subjective stress. Psychosom Med. 1979;41(3):209–18. 472086 10.1097/00006842-197905000-00004

